# Reverse vaccinology and immunoinformatics-driven design of a multiepitope vaccine candidate against *Renibacterium salmoninarum*

**DOI:** 10.3389/fbinf.2026.1899416

**Published:** 2026-09-25

**Authors:** Cristóbal Morales, Vicente Arriagada, Erick Bonilla, Bruno Prina, Esteban Godoy, Benjamín Molina-Chávez, Alberto Osorio, Emilio Gutierrez, Victor Roa, Leonardo A. Gómez, Ángel A. Oñate

**Affiliations:** 1 Laboratory of Molecular Immunology, Department of Microbiology, Faculty of Biological Sciences, University of Concepción, Concepción, Chile; 2 Simes Educational Center, Santiago, Chile

**Keywords:** bacterial kidney disease, immunoinformatics, molecular docking, molecular dynamics simulation, multiepitope vaccine, Renibacterium salmoninarum, reverse vaccinology, salmon aquaculture

## Abstract

*Renibacterium salmoninarum*, the etiological agent of bacterial kidney disease (BKD), remains a major challenge for salmonid aquaculture due to its intracellular lifestyle, chronic infection pattern, and the limited efficacy of current control strategies. In this study, a multiepitope vaccine candidate against *Renibacterium salmoninarum* was designed using reverse vaccinology and immunoinformatics approaches. Conserved antigenic proteins shared among three bacterial strains were identified through a systematic screening pipeline that included virulence prediction, essentiality analysis, antigenicity assessment, and exclusion of host homologs. Selected B-cell, MHC-I, and MHC-II epitopes were assembled into a chimeric construct and combined with conserved flagellin D0/D1 domains as a TLR5-targeting molecular adjuvant. The resulting construct, designated RS-MEVC, exhibited favorable predicted physicochemical and immunological properties, including high antigenicity, non-allergenicity, structural stability, and favorable stereochemical quality. Three-dimensional modeling revealed a predominantly α-helical structure, and structural validation supported the reliability of the refined model. Molecular docking predicted a stable interaction between RS-MEVC and *Atlantic salmon* TLR5, supported by multiple hydrogen bonds, salt bridges, and extensive intermolecular contacts. Molecular dynamics simulations further indicated stable conformational behavior of both the free vaccine construct and the vaccine-TLR5 complex, while MM-PBSA analysis predicted favorable binding energetics. Codon optimization and *in silico* cloning supported the feasibility of recombinant expression in *Escherichia coli*. Overall, these findings identify RS-MEVC as a promising computationally designed vaccine candidate against BKD and provide a rational framework for its subsequent experimental evaluation in salmonids.

## 1 Introduction

*Renibacterium* *salmoninarum* is a Gram-positive, non-motile, non-spore-forming coccobacillus that typically appears in pairs or short chains (Sanders and Fryer, 1980). It is a facultative intracellular pathogen capable of surviving and replicating within phagocytic cells, particularly macrophages, allowing it to evade host immune responses and limiting the effectiveness of conventional antimicrobial therapies (Bandín et al., 1995; Gutenberger et al., 1997; Grayson et al., 2002). This bacterium is the etiological agent of bacterial kidney disease (BKD), a chronic infection representing a major health challenge for salmonid aquaculture worldwide (Evenden et al., 1993; Wiens and Kaattari, 2019; Delghandi et al., 2020). BKD is characterized by a persistent and difficult to eradicate course, affecting both intensive farming systems and wild fish populations (Fryer and Sanders, 1981; Sakai and Kobayashi, 1992; Chambers et al., 2008). Clinically, infected fish may exhibit exophthalmia, skin lesions, hemorrhages at the base of fins, and systemic damage involving organs such as the kidney, liver, spleen, and heart (Grayson et al., 2001; Boerlage et al., 2017; Suzuki et al., 2017). From an immunological perspective, the kidney, the primary hematopoietic organ in fish, is severely compromised during infection, contributing to increased susceptibility to opportunistic pathogens (Bruneau et al., 1999; Zapata et al., 2006). The disease can affect all life stages, from juveniles to adults, and may reach mortality rates of up to 80% in Pacific salmon and approximately 40% in Atlantic salmon (*Salmo salar*) (Fairgrieve et al., 2005; Larson et al., 2020). However, BKD often presents as a chronic and subclinical infection, complicating its detection and control (Boerlage et al., 2017; Suzuki et al., 2017). Additionally, *Renibacterium salmoninarum* can be vertically transmitted and exhibits immunosuppressive mechanisms associated with the major virulence factor p57, promoting persistence and chronic infection (Wiens and Dale, 2009; Aguilar et al., 2023). This contributes to increased susceptibility to coinfections and negatively impacts production parameters such as growth rate and condition factor, particularly under low-temperature conditions (Wiens et al., 1999; Rozas-Serri et al., 2020). BKD has a global distribution, with reports across major salmon-producing regions including Europe, North America, Chile, Iceland, and Japan (Bayliss et al., 2018; Ahmed et al., 2025). While the disease has historically shown low prevalence in countries such as Norway, recent increases in outbreak frequency suggest a potential re-emergence scenario (Jansen and Silva de Oliveira, 2022). In contrast, prevalence in wild populations remains relatively low in recent surveys (Madhun et al., 2025). Collectively, these factors highlight the global relevance of BKD and the urgent need for more effective and sustainable preventive strategies.

Salmon aquaculture has become a cornerstone of global food production, a market valued at over USD 20 billion annually (FAO, 2022). Chile, the second-largest producer worldwide, contributes significantly to this industry, accounts for approximately 1.7%–2% of the national GDP and producing over 1.1 million tons annually (Subsecretaría de Pesca y Acuicultura, 2025). However, economically important species such as *S. salar*, *Oncorhynchus mykiss*, and *Oncorhynchus kisutch* are highly susceptible to *R. salmoninarum*, making BKD a critical threat to production (Flaño et al., 1996; Halaihel et al., 2009; Jaramillo et al., 2017). In Chile, the pathogen has been responsible for a substantial proportion of infectious mortality, with economic losses likely underestimated due to indirect impacts on growth performance, feed conversion, and overall productivity (SERNAPESCA, 2025). Currently, BKD control relies heavily on antimicrobial treatments, particularly during the freshwater stage (Fairgrieve et al., 2005). This approach increases production costs and contributes to the emergence of antimicrobial resistance, contrary to global efforts to mitigate this public health concern (Rhodes et al., 2008). Despite extensive research, no commercial vaccine has demonstrated consistent and long-lasting protection against *R. salmoninarum* under field conditions (Rhodes et al., 2004; Wiens, 2011). Traditional vaccine approaches, including inactivated and attenuated formulations, have shown limited efficacy, partly due to the pathogen’s ability to modulate host immune responses (Grayson et al., 2002; Rhodes et al., 2004). Notably, these strategies predominantly induce humoral responses, with limited capacity to elicit Th1-type cellular immunity, which is essential for controlling intracellular pathogens (Yamasaki et al., 2014; Valenzuela-Aviles et al., 2022).

In this context, immunoinformatics and genomics have emerged as powerful tools for the development of next-generation vaccines. These approaches enable the identification of relevant antigens, the optimization of vaccine constructs, and the rational targeting of immune responses. However, *in silico*-designed multiepitope vaccines often exhibit limited intrinsic immunogenicity, necessitating the incorporation of molecular adjuvants (Malonis et al., 2020; Saldanha et al., 2023; Zhao et al., 2023). In fish, activation of pattern recognition receptors such as Toll-like receptors (TLRs) plays a central role in initiating protective immune responses (Ghani et al., 2024). In the present study, a multiepitope vaccine candidate against *R. salmoninarum* was designed using a reverse vaccinology approach, incorporating conserved antigenic determinants and a flagellin-based adjuvant. The conserved D0/D1 domains of flagellin were selected because they contain the structural motifs required for recognition by Toll-like receptor 5 (TLR5), a key pattern-recognition receptor involved in the initiation of innate immune responses. Activation of TLR5 promotes the production of pro-inflammatory cytokines, including IL-6 and TNF-α, and enhances antigen presentation, thereby supporting the development of effective adaptive immune responses (Fields et al., 2024; Mardanova and Ravin, 2025). This approach is particularly relevant in salmonids, where TLR5 is structurally and functionally conserved and plays an important role in host defense against microbial pathogens (Tsujita et al., 2004; Wangkahart et al., 2019; Afzal et al., 2025). Furthermore, the D0/D1 domains retain potent immunostimulatory activity while allowing the incorporation of heterologous peptide sequences at their terminal regions without compromising receptor recognition, making flagellin an attractive platform for the development of recombinant multiepitope vaccines (Biedma et al., 2019). By promoting innate immune activation and supporting Th1-oriented cellular responses, which are essential for controlling intracellular bacterial infections, flagellin-based vaccine platforms may improve the efficacy of subunit vaccines against *R. salmoninarum*. Therefore, the objective of this study was to design and evaluate the structural, physicochemical, and immunogenic properties of a multiepitope vaccine candidate against *R. salmoninarum* using a reverse vaccinology approach.

## 2 Materials and methods

### 2.1 Identification and selection of candidate proteins

The selection of candidate proteins was initiated using the reference proteome of *R*. *salmoninarum* retrieved from the NCBI database (accession number GCF_000018885.1). To increase the representativeness of the vaccine design, proteomes from two Chilean strains, DJ2R and H2 (GCF_003343265.1 and GCF_003343285.1, respectively), were also included. The overall workflow used for the identification and selection of candidate proteins and subsequent vaccine design is illustrated in Figure 1. In the first step, subcellular localization of all proteins was predicted using PSORTb v3.0 (Yu et al., 2010), retaining proteins predicted to localize to the extracellular space, cell wall, cytoplasm, or cytoplasmic membrane, as these compartments are most relevant for vaccine antigen accessibility. Proteins with unknown localization were excluded. The selected proteins were then screened for similarity to experimentally validated virulence factors using the Virulence Factor Database (VFDB) (Liu et al., 2022). Proteins passing this filter were further analyzed against the Database of Essential Genes (DEG 15) (Luo et al., 2021) to identify those critical for pathogen survival. To minimize the risk of cross-reactivity with the host (*S. salar*), homology analysis was performed using BLASTp (Hu and Kurgan, 2019), retaining only proteins with pairwise sequence identity below 35% and an E-value threshold of 1 × 10^−10^. Transmembrane domains were predicted using TMHMM v2.0 (Krogh et al., 2001), excluding proteins containing more than one transmembrane helix. Finally, antigenicity was evaluated using VaxiJen v2.0 (Doytchinova and Flower, 2007), retaining proteins with scores ≥0.5. Conserved proteins shared among the three proteomes were selected to generate a consensus dataset for downstream epitope prediction.

**FIGURE 1:**
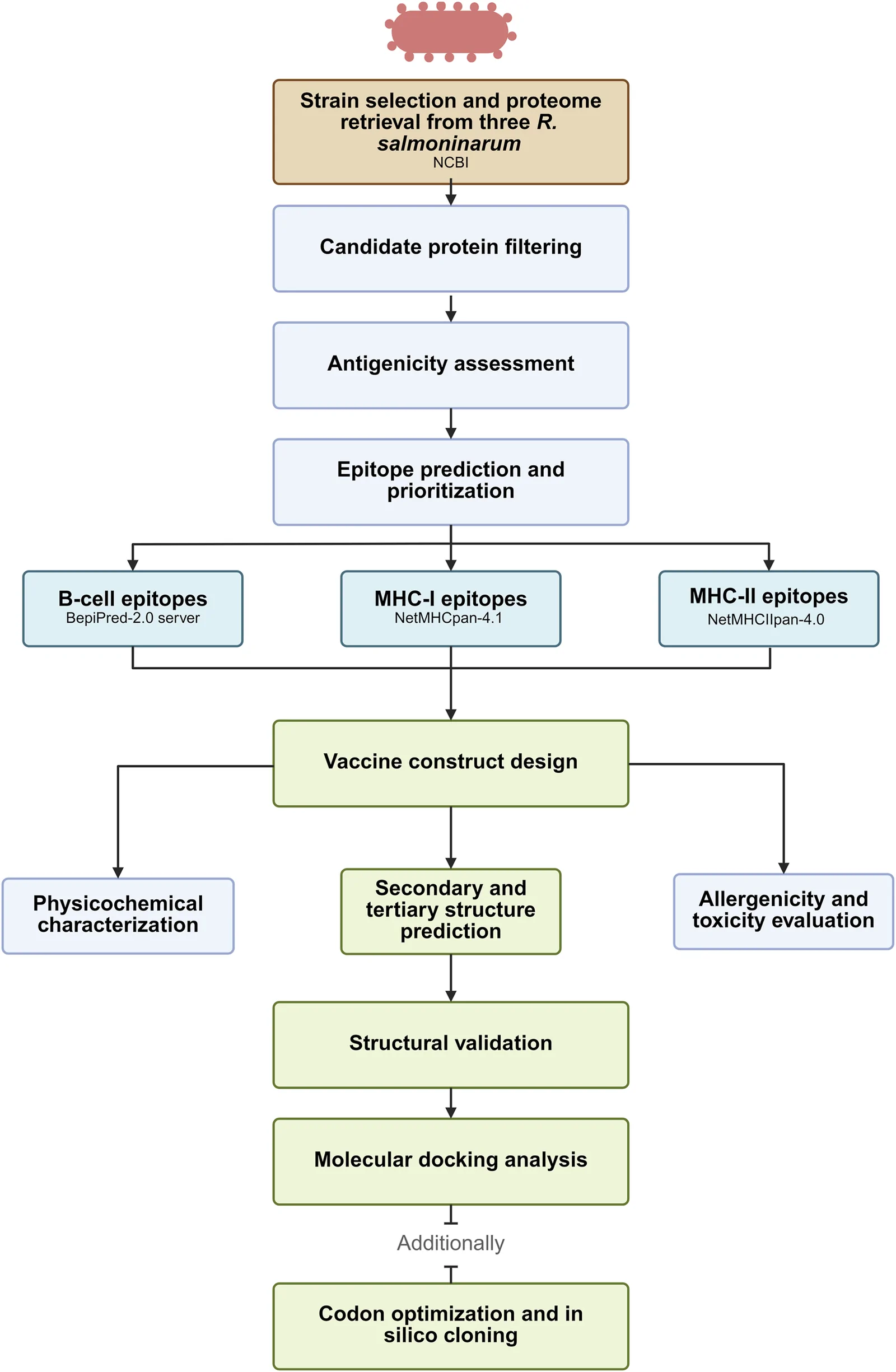
Stepwise screening workflow used to identify candidate vaccine proteins from three *Renibacterium salmoninarum* references proteomes. Protein candidates were sequentially filtered based on subcellular localization, virulence association, essentiality, lack of homology to the host, absence of transmembrane domains, antigenicity, and conservation across strains, resulting in 15 proteins selected for epitope prediction.

### 2.2 Prediction of B-cell epitopes

Linear B-cell epitopes were predicted using the BepiPred-3.0 server with the default parameters, using the previously selected consensus proteins as input (Clifford et al., 2022). Candidate epitopes identified by BepiPred were subsequently evaluated using the additional and structural criteria described below for downstream selection.

### 2.3 Prediction of MHC-I and MHC-II epitopes

For T-cell epitope prediction, the phylogenetic divergence between *S. salar* and the mammalian model organisms represented in standard MHC prediction databases was considered. MHC class I (CD8^+^ T-cell) epitopes were predicted using NetMHCpan-4.1, whereas MHC class II (CD4^+^ T-cell) epitopes were predicted using NetMHCIIpan-4.0 (Jurtz et al., 2017; Reynisson et al., 2020). These tools employ pan-specific models that incorporate MHC sequence information as an input, allowing peptide–MHC binding predictions based on sequence characteristics of the MHC molecule, including residues contributing to the peptide-binding groove. Therefore, MHC-I and MHC-II sequences from *S. salar* were used as model inputs rather than relying exclusively on predefined mammalian alleles. Teleost-specific or fish-adapted prediction tools were also considered; however, no suitable tool was identified for the specific MHC sequences analyzed in this study. The use of these predictors in fish species has previously been reported (Forouharmehr et al., 2022; Moin et al., 2024). Given the limited availability of experimentally characterized peptide–MHC binding datasets for salmonids, the resulting predictions were used as an *in silico* prioritization step rather than as experimental evidence of peptide–MHC binding.

Allelic data was retrieved from the IPD database. For MHC class I, 48 classical Sasa-UBA alleles were retrieved, of which 35 could be used for prediction because a valid peptide-binding pseudo-sequence could not be generated for the remaining 13 alleles. Subsequent MHC-I promiscuity analyses were therefore performed using the 35 alleles for which valid predictions were obtained. For MHC class II, the heterodimeric receptor was represented by the retrieved DAA (α-chain) and DAB (β-chain) variants. All possible combinations of the 22 DAA and 42 DAB variants were generated, resulting in 924 DAA/DAB heterodimer combinations, and each candidate epitope was evaluated against these combinations. A Score_EL ≥ 0.6 was used as the threshold for considering a predicted peptide–MHC interaction. To maximize theoretical population coverage, epitopes were prioritized based on promiscuity, defined as their predicted ability to bind multiple MHC variants, quantified as the number of alleles or DAA/DAB heterodimer combinations for which the Score_EL was ≥0.6.

### 2.4 Construct design

The five highest-scoring epitopes from each category (B-cell, MHC-I, and MHC-II) were selected for construction of the multiepitope vaccine. A conserved flagellin D0/D1 inner core domain, shared among diverse bacterial species, was incorporated as a molecular adjuvant. The D0 and D1 domains were incorporated while preserving their native arrangement, with the D0/D1 region positioned at the N-terminus and the D1/D0 region at the C-terminus of the construct (Biedma et al., 2019). These domains were connected using rigid EAAAK linkers. Epitopes were joined using linker sequences according to their category: KK linkers for B-cell epitopes, AAY linkers for MHC-I epitopes, and GPGPG linkers for MHC-II epitopes. These linkers were included to facilitate efficient proteolytic processing and antigen presentation (Chen et al., 2013; Ayyagari et al., 2022). *BamH*I and *Sal*I restriction sites were incorporated at the 5′ and 3′ ends of the optimized RS-MEVC coding sequence, respectively, to facilitate directional cloning into the expression vector. The final coding sequence was screened to confirm the absence of internal *BamH*I and *Sal*I restriction sites.

### 2.5 Physicochemical properties

Physicochemical properties of the construct were evaluated using ProtParam (Wilkins et al., 1999), including molecular weight, theoretical isoelectric point (pI), instability index, aliphatic index, and GRAVY score in order to assess the physicochemical feasibility and stability of the vaccine construct. Antigenicity was reassessed using VaxiJen, while allergenicity was predicted using AllerTOP v2.0 (Dimitrov et al., 2014), and toxicity was evaluated using ToxinPred2 (Sharma et al., 2022), to further assess the safety, stability, and suitability of the vaccine construct.

### 2.6 Secondary structure analysis

Secondary structure was analyzed using PDBsum (Laskowski et al., 2018), based on the PROMOTIF algorithm (Hutchinson and Thornton, 1996). This analysis aimed to characterize the proportion of ordered structural elements, including α-helices and β-strands, which are critical determinants of protein folding and stability.

### 2.7 Tertiary structure prediction, refinement, and validation

The three-dimensional structure was predicted using AlphaFold3 (Abramson et al., 2024), selected for its high accuracy in modeling complex protein structures. The predicted model was visualized using PyMOL (Schrödinger LLC, 2015) and refined at the energetic and topological levels using GalaxyRefine (Heo et al., 2013). Structural quality was assessed using MolProbity (Williams et al., 2018), evaluating covalent geometry and atomic contacts. Additional validation was performed using ProSA-web (Wiederstein and Sippl, 2007), PROCHECK (Laskowski et al., 1993), and ERRAT (Colovos and Yeates, 1993), confirming appropriate stereochemical quality. The final model was considered suitable for downstream molecular docking and molecular dynamics simulations with immune receptors of *S. salar*, such as TLR5.

### 2.8 Molecular docking

To predict protein–protein interactions, molecular docking was performed using ClusPro 2.0 (Jones et al., 2022). This platform was selected as a suitable restraint-free protein–protein docking approach for the present analysis because the RS-MEVC construct represents a novel chimeric protein for which experimentally determined interaction restraints were not available. HADDOCK and ZDOCK were considered as alternative docking approaches. ClusPro was selected because it performs extensive rigid-body sampling followed by clustering and scoring of candidate docking solutions, allowing candidate complexes to be generated without requiring predefined interaction residues. The approach has also demonstrated strong performance in the Critical Assessment of Predicted Interactions (CAPRI) blind trials (Jones et al., 2022).

The tertiary structure of *S. salar* TLR5 was retrieved from the AlphaFold Protein Structure Database under the entry AF-F5B7Y2-F1 (Varadi et al., 2024). Multiple docking solutions generated by ClusPro were evaluated based on the clustering and scoring criteria provided by the server. The representative pose from the most populated, lowest-energy cluster was selected for subsequent structural and interaction analyses. The resulting docked complex was analyzed using the PDBsum web server to characterize the predicted interaction interface, including hydrogen bonds, salt bridges, and non-bonded contacts. Following the same procedure, the experimentally determined TLR5–flagellin complexes PDB 1U0A and 3V47 were analyzed using PDBsum for comparison. Because these experimental structures contain higher-order assemblies, the primary 1:1 TLR5–flagellin recognition interface was isolated before analysis to enable a method-consistent comparison with the predicted 1:1 TLR5–RS-MEVC complex. This comparison was used to assess whether the interaction features predicted for RS-MEVC were consistent with those observed in experimentally characterized TLR5–flagellin complexes.

### 2.9 Molecular dynamics simulation

Molecular dynamics simulations (MDSs) were performed to evaluate the structural stability of the refined vaccine construct in both its free form and in complex with TLR5. The simulation workflow consisted of the following steps.

- Ensemble preparation: The CHARMM-GUI server was used to generate GROMACS v2025.1 input file through the Solution Builder module. The systems were solvated in cubic boxes using the TIP3P water model (Brooks et al., 2009; Lee et al., 2016), maintaining a minimum distance of 10 Å between the protein surface and the box edges. The free vaccine construct was placed in a cubic box with a side length of 203 Å, whereas the vaccine-TLR5 complex was solvated in a cubic box with a side length of 201 Å. K^+^ and Cl^−^ ions were added using Monte Carlo ion placement until electrical neutrality was achieved. Both systems were parameterized using the Amber force field (ff19SB) (Lee et al., 2020). Long-range electrostatic interactions were calculated using the Particle Mesh Ewald (PME) method with a real-space Coulomb cutoff of 0.9 nm. Van der Waals interactions were treated using a Verlet cutoff scheme with a cutoff distance of 0.9 nm.
- Energy minimization: The potential energy of each system was minimized using the steepest descent algorithm.
- Equilibration: Equilibration was performed under NVT and NPT ensembles at 303.15 K and 1 bar pressure. Temperature coupling was maintained using the velocity-rescale (v-rescale) thermostat, whereas pressure coupling was controlled isotropically using the c-rescale barostat.
- Production run: Production simulations were carried out for 100 ns using a 2 fs integration time step. System energies were recorded every 10 ps.

To assess the conformational stability of both the vaccine construct and the vaccine–TLR5 complex, root mean square deviation (RMSD) of backbone atoms, radius of gyration (Rg), and root mean square fluctuation (RMSF) of Cα atoms were analyzed.

To account for the temporal autocorrelation inherent to molecular dynamics trajectories, which precludes treating individual frames as independent samples (Grossfield and Zuckerman, 2009), RMSD and Rg were quantified over the equilibrated portion of each simulation (t > 10 ns). Statistical uncertainty was estimated using autocorrelation-corrected 95% confidence intervals obtained by Flyvbjerg–Petersen block averaging (Flyvbjerg and Petersen, 1989). Formal hypothesis testing was not applied to RMSD or Rg because these metrics represent single temporally correlated trajectories rather than independent replicate observations. For RMSF, Cα fluctuations were compared between the free and bound states across the 422 corresponding residue positions using paired t-test and Wilcoxon signed-rank test (Wilcoxon, 1945), with Cohen’s dz reported as an effect-size measure.

Binding free energies of the docked complexes were estimated using the molecular mechanics Poisson–Boltzmann surface area (MM-PBSA) method (Huo et al., 2011). Calculations were performed using the gmx_MMPBSA tool (Valdés-Tresanco et al., 2021), using the last 200 frames of the production trajectory.

### 2.10 Codon optimization and *in silico* cloning of the vaccine construct

Codon optimization is essential for efficient heterologous gene expression in host organisms (Al-Hawash et al., 2017). The vaccine construct sequence was reverse-translated and codon-optimized for *Escherichia coli* (strain K-12) using the EMBOSS Backtranseq tool (Madeira et al., 2019). The optimized sequence was further evaluated using the GenScript Rare Codon Analysis tool (https://www.genscript.com/tools/rare-codon-analysis/) to assess codon usage bias and predict expression efficiency. In silico cloning of the optimized construct was performed using SnapGene software (Samad et al., 2020). The pET-28a (+) expression vector, retrieved from the Addgene repository (Kamens, 2015), was used as the cloning backbone. This vector includes key regulatory elements such as the T7 promoter, Shine–Dalgarno sequence, and translation initiation codon within the expression cassette. The optimized construct sequence was modified at the 5′ and 3′ ends to include *BamHI* and *SalI* restriction sites, as well as a stop codon, ensuring correct insertion into the vector and maintenance of the proper reading frame. The bioinformatic tools, databases, and web servers used throughout the study are summarized in Supplementary Table S1.

## 3 Results

### 3.1 Selection of candidate proteins from the *Renibacterium salmoninarum* proteome

Candidate protein selection was performed independently and in parallel using the proteomes of 3 *R. salmoninarum* strains: the reference strain ATCC 33209 (2,926 proteins; Figure 2A) and the field strains DJ2R (2,925 proteins; Figure 2B) and H2 (2,925 proteins; Figure 2C). Subcellular localization analysis revealed a highly similar distribution across all strains. In the reference strain, 1,327 proteins were predicted to be cytoplasmic, 892 associated with the cytoplasmic membrane, 57 extracellular, and 14 cell wall-associated, in addition to 636 proteins of unknown localization. Comparable distributions were observed for strains DJ2R and H2 (Figure 2D). Following this initial filtering step, 140 proteins were identified in the ATCC 33209 and DJ2R proteomes and 137 proteins in strain H2 based on similarity to known virulence factors. Subsequent screening against the Database of Essential Genes (DEG) reduced this set to 124 essential proteins per proteome. To minimize the risk of cross-reactivity with the host (*S. salar*), proteins showing significant homology to host sequences were excluded, resulting in 56 non-homologous proteins per proteome. Further filtering to remove proteins containing more than one transmembrane domain reduced the candidate pool to 39 proteins per proteome.

**FIGURE 2:**
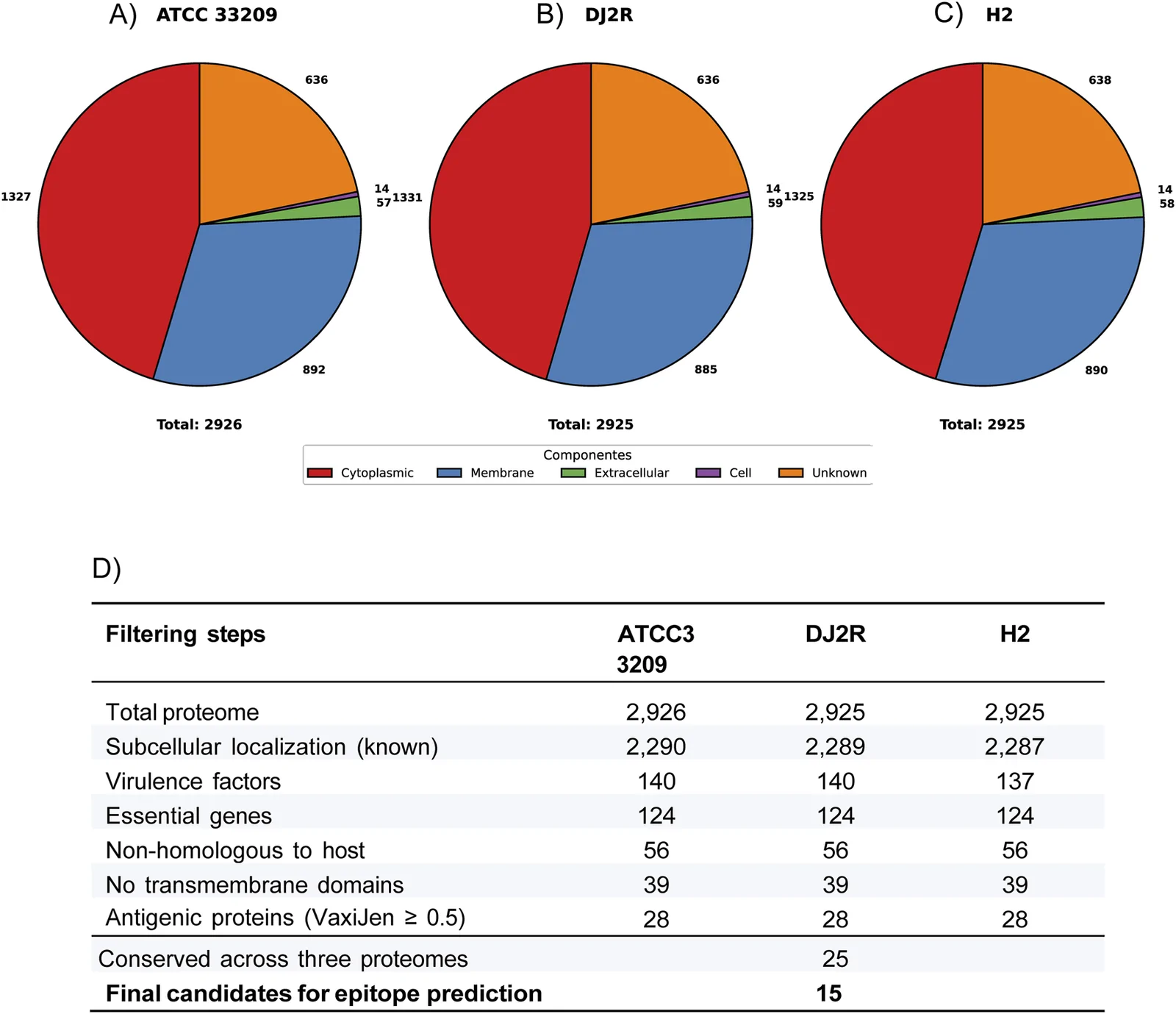
Predicted subcellular localization distribution of the proteome from three reference strains of *Renibacterium salmoninarum*. **(A)** Strain ATCC 33209, comprising 2,926 predicted proteins. **(B)** Strain DJ2R, comprising 2,925 predicted proteins. **(C)** Strain H2, comprising 2,925 predicted proteins. **(D)** Summary of the sequential filtering steps applied to the three proteomes, yielding 15 candidate proteins for epitope prediction. In all strains, the majority of proteins were classified as cytoplasmic and membrane-associated, while a smaller proportion corresponded to extracellular, cell surface-associated, and proteins with unknown subcellular localization.

Finally, antigenicity prediction identified 28 antigenic proteins per proteome. Comparative analysis across the three datasets revealed 25 consensus proteins shared among all strains. From this group, the 15 proteins with the highest antigenicity scores (range: 0.5321–0.6932) were selected for downstream epitope prediction (Table 1).

**TABLE 1:** Characteristics of the 15 candidate proteins selected from the *Renibacterium salmoninarum* proteome for multi-epitope vaccine design.

Protein ID	Subcellular localization (PSORTb)	Molecular weight (Da)	Transmembrane helices	Topology	Antigenicity score
WP 012245734.1 Imidazole glycerol phosphate synthase subunit HisF	Cytoplasmic	26,626.16	0	Globular	0.6932
WP 041684574.1 MtrAB system response regulator MtrA	Cytoplasmic	24,729.66	0	Globular	0.6332
WP 233494209.1 Bifunctional UDP-N-acetylglucosamine diphosphorylase/ glucosamine-1-phosphate N-acetyltransferase GlmU	Cytoplasmic	50,510.73	0	Globular	0.6310
WP 012243730.1 Gamma carbonic anhydrase family protein	Cytoplasmic	17,312.42	0	Globular	0.6235
WP 012244355.1 response regulator	Cytoplasmic	24,823.22	0	Globular	0.6090
WP 012246528.1 M4 family metallopeptidase	Cell wall	56,378.64	0	Globular	0.5986
WP 012243940.1 response regulator transcription factor	Cytoplasmic	26,781.36	0	Globular	0.5944
WP 041684261.1 response regulator transcription factor	Cytoplasmic	25,350.13	0	Globular	0.5895
WP 233494250.1 C40 family peptidase	Extracellular	20,265.72	0	Globular	0.5865
WP 049758741.1 isocitrate lyase	Cytoplasmic	40,419.14	0	Globular	0.5848
WP 012243656.1 sensor histidine kinase	Cytoplasmic membrane	24,636.80	0	Globular	0.5543
WP 012245978.1 ParA family protein	Cytoplasmic membrane	31,570.16	0	Globular	0.5523
WP 012243868.1 response regulator transcription factor	Cytoplasmic	25,878.66	0	Globular	0.5440
WP 233494262.1 imidazole glycerol phosphate synthase subunit HisH	Cytoplasmic	23,335.56	0	Globular	0.5384
WP 012244415.1 LuxR C-terminal-related transcriptional regulator	Cytoplasmic	22,353.40	0	Globular	0.5321

### 3.2 Prediction of B-cell and T-cell (MHC-I and MHC-II) epitopes and construct design

From the 15 selected proteins, a total of 75 linear B-cell epitopes (9–15 amino acids in length) were initially predicted using BepiPred. Following antigenicity evaluation, the five epitopes with the highest scores (0.6981–0.8053) were selected (Table 2). For MHC class I epitope prediction, binding affinity was evaluated using the classical Sasa-UBA locus of *S. salar*. Of the 48 UBA alleles retrieved from the IPD database, 35 could be used for prediction, as a valid peptide-binding pseudo-sequence could not be generated for the remaining 13. A total of 25 epitopes exhibited a Score_EL ≥ 0.6 in at least one allele, with allele coverage ranging from one to seven alleles per epitope (2.1%–14.6%). From this set, the five epitopes with the highest antigenicity were selected (antigenicity scores: 0.7543–1.5127; Score_EL: 0.637–0.793). Similarly, for MHC class II, 25 epitopes with the highest binding scores were identified against the 924 DAA/DAB heterodimer combination generated from 42 DAB and 22 DAA alleles. From these, the five most antigenic epitopes were selected (antigenicity scores: 0.9503–1.4213; mean Score_EL: 0.686–0.810) (Table 2). Based on these results, the final construct, designated RS-MEVC, was designed as a 422-amino acid sequence with a modular organization. The construct incorporates adjuvant domains derived from flagellin due to their ability to activate Toll-like receptor 5 (TLR5), a conserved receptor in salmonids, thereby enhancing innate immune signaling and promoting adaptive immune responses (Figure 3).

**TABLE 2:** Epitopes selected from candidate proteins of *Renibacterium salmoninarum* for inclusion in the multi-epitope vaccine construct.

Epitope class	Epitope	Length (aa)	Protein ID	Antigenicity	Mean binding score*
B	RQVTNEASAEDHNQS	15	WP_233494209.1	0.8053	—
	AQTVTASKDANI	12	WP_233494209.1	0.8030	—
	GPTGRPMTEFPE	12	WP_012245978.1	0.7942	—
	SNTAQAKAPAEP	12	WP_233494250.1	0.7671	—
	AAQTVTASKDAN	12	WP_233494250.1	0.6981	—
MHC I	RISLTPLEF	9	WP_041684574.1	1.5127	0.670
	MKPPAVTW	9	WP_233494262.1	1.0327	0.793
	EPDPSSPRY	9	WP_012243868.1	1.0084	0.637
	RVRLTPTEW	9	WP_012244355.1	0.9153	0.709
	VAAEPITTY	9	WP_012245978.1	0.7543	0.706
MHC II	DTRLVNVHVQRLRSK	15	WP_041684574.1	1.4213	0.767
	ADTRLVNVHVQRLRS	15	WP_041684574.1	1.2649	0.686
	DEELEGIRSNAAHYR	15	WP_012243730.1	1.1599	0.810
	SDEELEGIRSNAAHY	15	WP_012243730.1	0.9602	0.740
	RNNFEGEAADKADVV	15	WP_041684261.1	0.9503	0.689

*Mean binding scores correspond to the average EL score (score_EL) across all evaluated alleles. Not applicable (--) for Class B epitopes.

**FIGURE 3:**
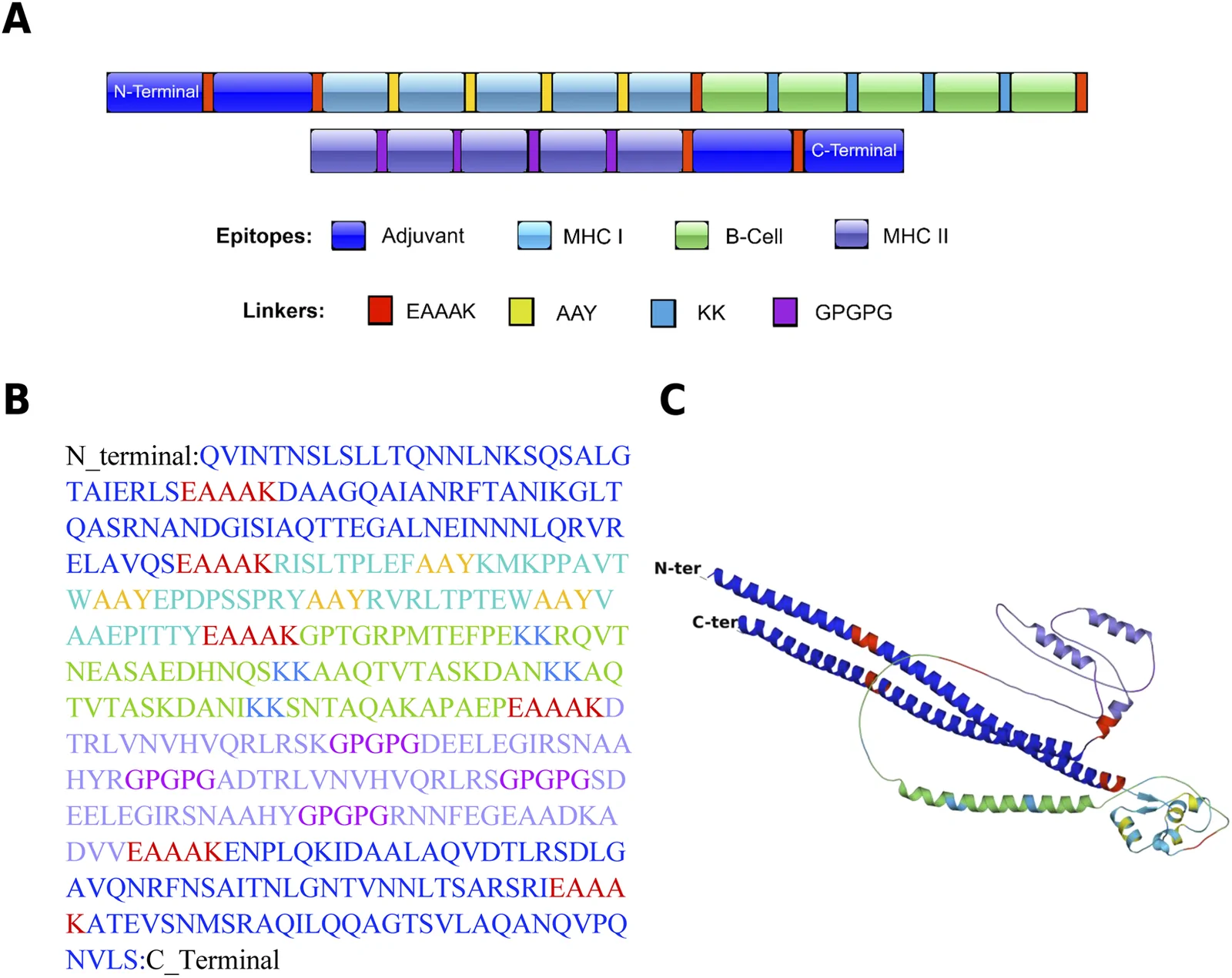
Schematic representation of the RS-MEVC multiepitope vaccine construct. **(A)** The modular organization of the construct includes N- and C-terminal flagellin-derived adjuvant domains (blue), EAAAK rigid linkers (orange), five MHC class I epitopes (cyan) separated by AAY linkers (yellow), five B-cell epitopes (green) separated by KK linkers (light blue), and five MHC class II epitopes (lavender) separated by GPGPG linkers (purple). **(B)** Complete amino acid sequence of the construct, color-coded by segment as in **(A)**, from the N-terminus to the C-terminus. **(C)** Predicted three-dimensional (ribbon) model of the construct, with the N- and C-terminal ends indicated. The final construct consisted of 422 amino acids.

### 3.3 Design of the multiepitope vaccine

The construct, designated the *R. salmoninarum* Multi Epitope Vaccine Construct (RS-MEVC), was designed as a 422-amino acid chimeric protein with a modular organization. MHC-I, B-cell, and MHC-II epitopes were assembled sequentially and joined using their corresponding linkers. The epitope region was flanked at both the N- and C-termini by the D0/D1 domains of flagellin, which were incorporated as a molecular adjuvant (Figures 3A,B). The conserved D0/D1 domains were positioned at both termini of the construct to flank the antigenic region and preserve the structural elements required for TLR5-mediated immunostimulation. The overall architecture and structural organization of the construct were further verified by visual inspection using PyMOL (Figure 3C).

### 3.4 Physicochemical properties

Physicochemical analysis indicated that RS-MEVC has a molecular weight of 44,737.66 Da, a theoretical isoelectric point (pI) of 9.26, and a GRAVY score of −0.606, suggesting a hydrophilic nature. The instability index was 37.35, classifying the construct as stable, and the aliphatic index was 74.45, indicating potential thermostability. The construct exhibited an overall antigenicity score of 0.7723 and was predicted to be non-allergenic, supporting its suitability as a vaccine candidate for further structural and functional analyses (Table 3).

**TABLE 3:** Predicted physicochemical, immunological, and safety-related properties of the final construct RS-MEVC.

Property	Value
Number of amino acids	422
Molecular weight (Da)	44,737.66
Theoretical pI	9.26
Instability index	37.35 (stable)
Aliphatic index	74.45
Grand average of hydropathicity (GRAVY)	−0.606
Antigenicity (VaxiJen)	0.7723 (probable antigen)
Allergenicity (AllerTOP)	Probable non-allergen
Toxicity (ToxinPred2)	Predicted as non-toxic
Solubility (SOLpro)	0.52 (soluble)

### 3.5 Secondary structure and 3D structure prediction

Secondary structure analysis revealed a predominantly α-helical organization. The 422-residue construct consisted of 229 residues in α-helices (54.3%), 15 in 3_10_ helices (3.6%), 20 in β-strands (4.7%), and 158 residues in other conformations (37.4%) (Figure 4A). The three-dimensional structure was refined using the GalaxyRefine server, yielding models with global quality scores ranging from 90.2 to 99.5. The best-ranked model displayed a compact conformation dominated by α-helices, with well-defined domains connected by exposed loop regions, achieving a score of 99.5. This structural organization was further confirmed by visual inspection using PyMOL (Figure 4B).

**FIGURE 4:**
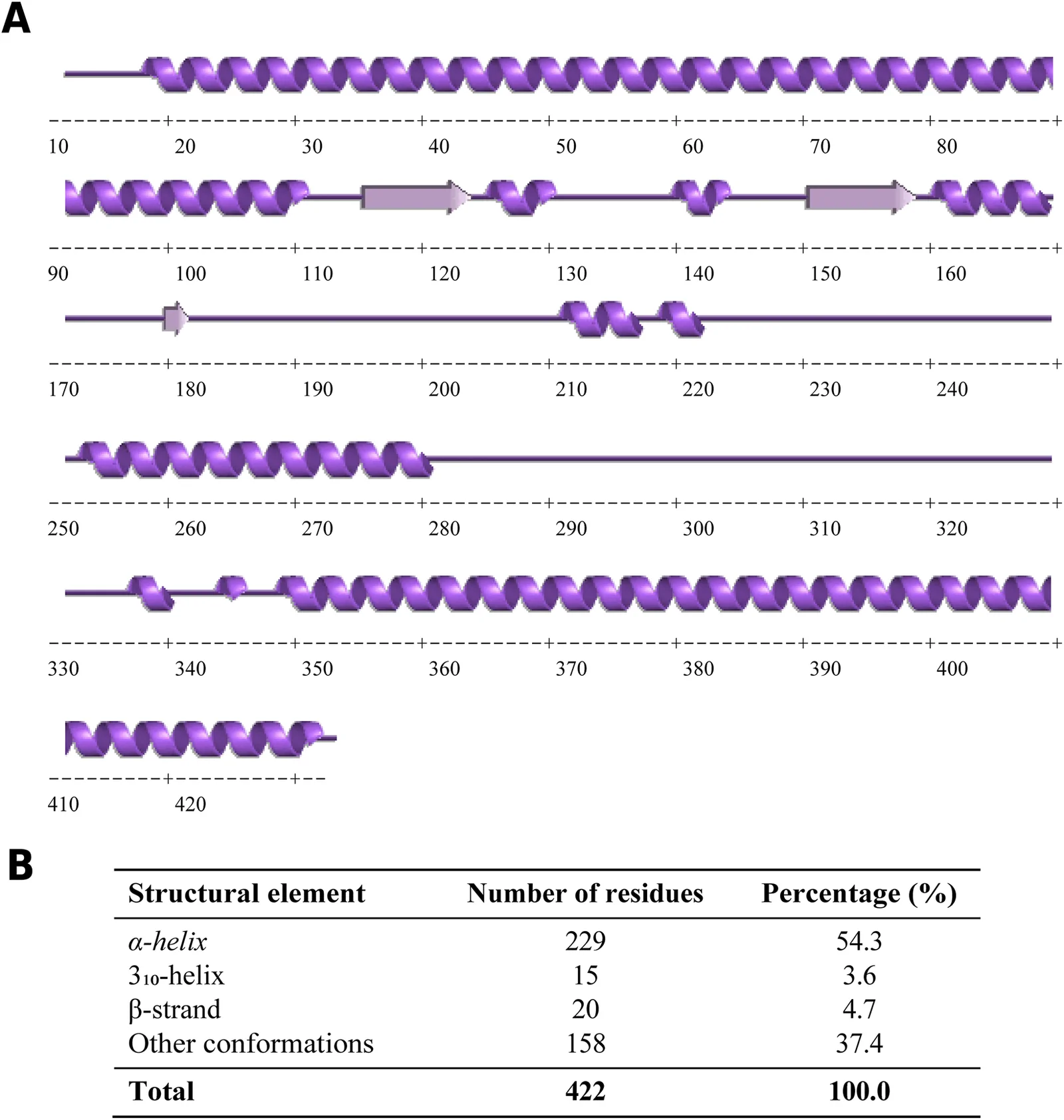
Predicted secondary structure of the RS-MEVC multiepitope vaccine construct. **(A)** structure along the 422-residue sequence (residue numbers in increments of ten), showing a predominance of α-helical elements with a lower proportion of β-strands and coil regions. **(B)** Distribution of secondary-structure elements: α-helix (229 residues, 54.3%), 3₁₀-helix (15 residues, 3.6%), β-strand (20 residues, 4.7%), and other conformations (158 residues, 37.4%), for a total of 422 residues.

### 3.6 Validation of the tertiary structure

Structural validation of the refined RS-MEVC model demonstrated high overall quality and reliability. Residue-level energy analysis performed using ProSA-web revealed predominantly negative energy values throughout the sequence (Figure 5A), indicating a favorable structural environment and the absence of major local structural anomalies. Furthermore, ProSA-web analysis yielded a Z-score of −5.92 (Figure 5B), placing the construct within the range typically observed for experimentally determined native proteins of similar size. The stereochemical quality of the refined model was further assessed using Ramachandran plot analysis. A total of 98.9% of residues were located in the most favored regions, while the remaining 1.1% were distributed within additionally allowed regions, with no residues detected in disallowed regions (Figure 5C). These results indicate excellent backbone geometry and structural integrity. Collectively, the ProSA and Ramachandran analyses support the overall quality, stability, and reliability of the refined RS-MEVC model, validating its suitability for subsequent molecular docking and molecular dynamics simulation studies (Figure 5).

**FIGURE 5:**
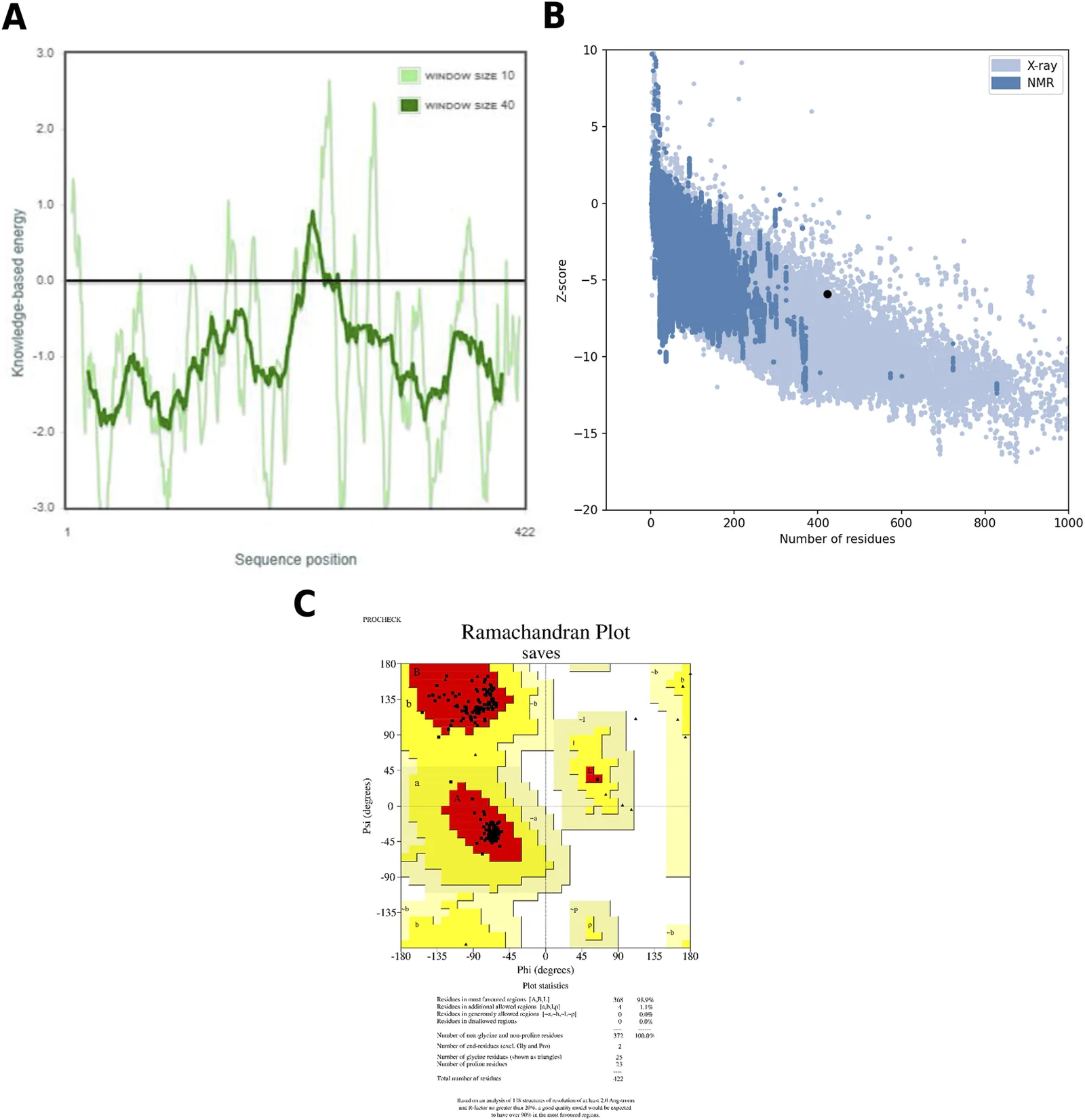
Structural quality validation of the RS-MEVC multiepitope vaccine construct. **(A)** Residue-wise energy profile predicted by ProSA along the sequence, showing predominantly negative values consistent with a structurally stable model. **(B)** Z-score plot obtained using ProSA, indicating that the model falls within the range characteristic of experimentally resolved protein structures. **(C)** Ramachandran plot generated by PROCHECK, showing that 98.9% of residues are located in the most favored regions.

### 3.7 Molecular docking analysis

Molecular docking was performed to evaluate the interaction between the RS-MEVC vaccine construct and Atlantic salmon TLR5. The resulting docking pose revealed a stable binding interface between the vaccine construct (chain B) and TLR5 (chain A) (Figure 6A). Analysis of the interaction network identified 15 hydrogen bonds, 5 salt bridges, and 206 non-bonded contacts at the protein-protein interface (Figure 6B), indicating extensive intermolecular interactions that may contribute to complex stability. The specific amino acid residues involved in hydrogen-bond formation are listed in Table 4. To further evaluate the stability of the predicted complex under physiological conditions, molecular dynamics simulations were subsequently performed for both the free vaccine construct and the vaccine-TLR5 complex.

**FIGURE 6:**
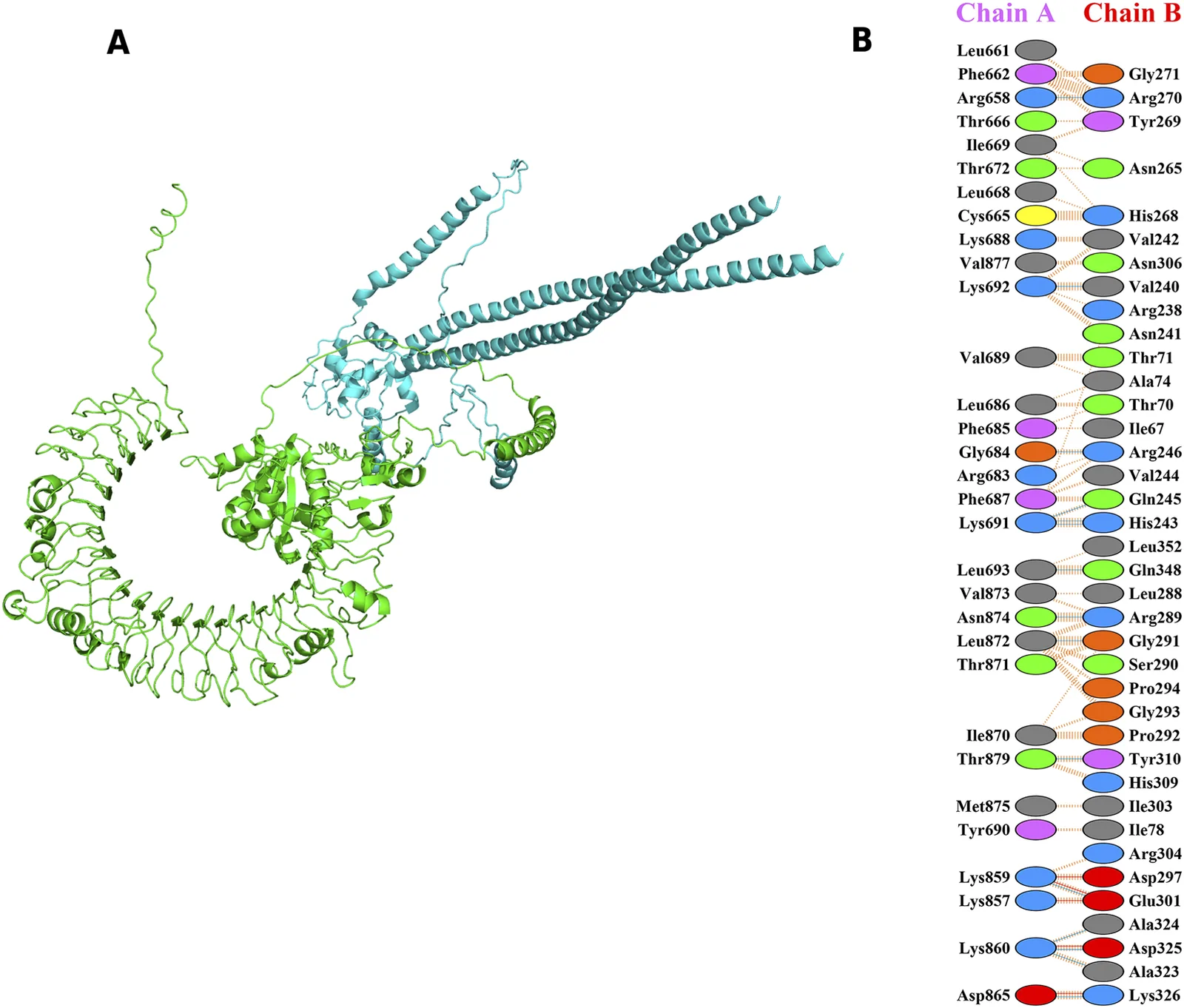
Molecular docking analysis of the RS-MEVC vaccine construct with *Atlantic salmon* TLR5. **(A)** Three-dimensional representation of the docking complex between the vaccine construct (cyan) and *Salmo salar* TLR5 (green). **(B)** Detailed view of the molecular interaction interface between chain A (TLR5) and chain B (vaccine construct).

**TABLE 4:** Hydrogen-bond interactions between chains A (TLR5) and B (vaccine construct).

Residue (A)	Res no. (A)	Chain (A)		Residue (B)	Res no. (B)	Chain (B)	Distance (Å)
ARG	658	A	< >	ARG	270	B	2.75
GLY	684	A	< >	ARG	246	B	2.84
LYS	691	A	< >	HIS	243	B	3.13
LYS	691	A	< >	HIS	243	B	2.84
LYS	691	A	< >	GLN	245	B	2.59
LYS	692	A	< >	VAL	240	B	2.54
LEU	693	A	< >	GLN	348	B	2.73
LYS	859	A	< >	GLU	301	B	2.76
LYS	860	A	< >	ALA	323	B	2.68
LYS	860	A	< >	ALA	324	B	2.51
LYS	860	A	< >	ASP	325	B	2.69
ASP	865	A	< >	LYS	326	B	2.53
LEU	872	A	< >	GLY	291	B	2.88
ASN	874	A	< >	ARG	289	B	2.68

### 3.8 Molecular dynamics simulation

Molecular dynamics simulations were conducted to assess the structural stability and dynamic behavior of the RS-MEVC vaccine construct in its free form and when bound to TLR5. To avoid pseudoreplication arising from the temporal autocorrelation of the trajectories, equilibrium metrics were quantified over the post-equilibration window, and statistical uncertainty was estimated using the Flyvbjerg–Petersen block-averaging method to yield autocorrelation-corrected standard errors and 95% confidence intervals (CIs).

Backbone RMSD analysis revealed an initial increase during the first 10 ns of simulation in both systems, corresponding to structural relaxation and equilibration. Following this phase, both systems maintained stable trajectories throughout the remainder of the simulation. Notably, the vaccine–TLR5 complex exhibited lower mean backbone RMSD than the free construct (1.32 nm, 95% CI [1.24, 1.41] versus 1.70 nm, 95% CI [1.58, 1.82]). The corresponding mean difference of 0.38 nm (95% CI [0.23, 0.53]), supporting a lower mean global backbone deviation in the complex over the analyzed trajectory (Figures 7A,B).

**FIGURE 7:**
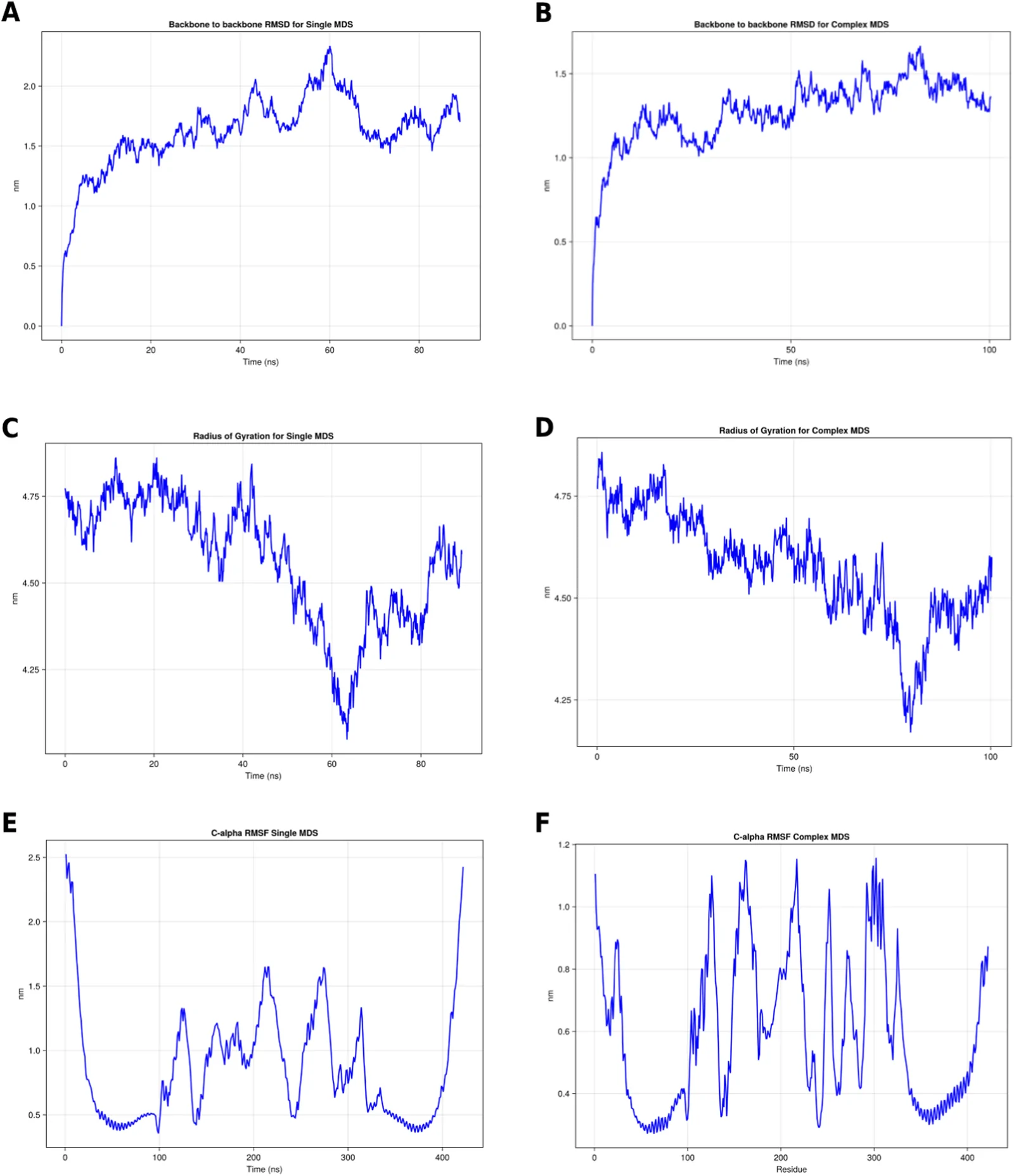
Molecular dynamics simulation analyses of the RS-MEVC vaccine construct in free and docked conformations. **(A)** Backbone RMSD profile of the free-form vaccine construct. **(B)** Backbone RMSD profile of the vaccine–TLR5 complex. **(C)** Radius of gyration (Rg) profile of the free-form vaccine construct. **(D)** Radius of gyration profile of the vaccine-TLR5 complex. **(E)** Cα RMSF profile of atoms in the free-form vaccine construct. **(F)** RMSF profile of Cα atoms in the vaccine-TLR5 complex.

The radius of gyration (Rg) remained comparable between the two systems throughout the equilibrated window (complex, 4.55 nm, 95% CI [4.46, 4.64]; free construct, 4.55 nm, 95% CI [4.40, 4.69]), with a mean difference of −0.01 nm (95% CI [−0.18, 0.16]) whose confidence interval encompassed zero. Both systems therefore maintained a similar overall degree of compactness, with no evidence of a difference in Rg between the free and bound states (Figures 7C,D).

Residue-level flexibility was assessed using RMSF analysis of Cα atoms. Because both systems comprise the same 422-residue construct, per-residue values were compared as paired observations. The free construct displayed pronounced fluctuations at terminal segments and selected internal loops, whereas the complex showed generally lower flexibility, with 377 of 422 residues (89%) exhibiting reduced RMSF upon binding. The mean per-residue reduction was 0.27 nm (95% CI [0.24, 0.30]; paired t = 16.4, Wilcoxon signed-rank p < 0.0001; Cohen’s dz = 0.80), indicating a significant reduction in per-residue fluctuation upon receptor association (Figures 7E,F).

Collectively, the RMSD and RMSF analyses indicate that TLR5 association is accompanied by reduced global backbone deviation and lower local residue fluctuations in RS-MEVC, while the unchanged radius of gyration indicates that these changes occur without a detectable alteration in overall compactness. Together, these findings are consistent with a structurally stable complex over the simulated timescale.

To further evaluate the strength of the interaction energetics, binding free energy calculations were performed using the MM-PBSA approach. The last 200 frames of the simulation were analyzed. The interaction was primarily driven by highly favorable electrostatic (−1,080.73 ± 87.78 kcal/mol) and van der Waals (−272.31 ± 16.71 kcal/mol) contributions, which compensated for the unfavorable solvation energy (1,187.20 ± 85.40 kcal/mol). The resulting negative MM-PBSA binding free energy (ΔG = −165.83 ± 12.47 kcal/mol) supports a favorable predicted interaction between the vaccine construct and TLR5 within the simulated system (Table 5). However, given the 100 ns simulation timescale, these results should be interpreted as evidence of short-term structural and energetic stability rather than definitive evidence of long-term complex integrity.

**TABLE 5:** Estimated binding-free energy for the vaccine construct–TLR5 complex.

Energy contributions	Construct-TLR5 complex (kcal/mol)
Δ electrostatic energy	−1,080.73 ± 87.78
Δ van de Waals energy	−272.31 ± 16.71
Δ gas phase energy	−1,353.03 ± 85.97
Δ solvation energy	1,187.20 ± 85.40
ΔG	−165.83 ± 12.47

### 3.9 Codon optimization and *in silico* cloning of the vaccine construct

The sequence optimized for *E. coli* K-12 resulted in a length of 1,266 base pairs, with a codon adaptation index (CAI) of 0.89 and a GC content of 59%. Although a CAI value above 0.90 is commonly considered favorable for high-level heterologous expression, the obtained value was close to this threshold. The GC content of the 59% was also relatively high for *E. coli*, representing a parameter that may potentially influence local mRNA secondary structure and translation efficiency. Using SnapGene software (version 8.2.2), the optimized construct was cloned *in silico* into the pET-28a (+) expression vector between the *Sal*I (position 179) and *BamH*I (position 198) restriction sites. The resulting recombinant pET-28a (+) plasmid containing the insert was 6,632 bp in length (Figure 8A). The cloning strategy was further validated by simulated agarose gel electrophoresis using SnapGene (Figure 8B).

**FIGURE 8:**
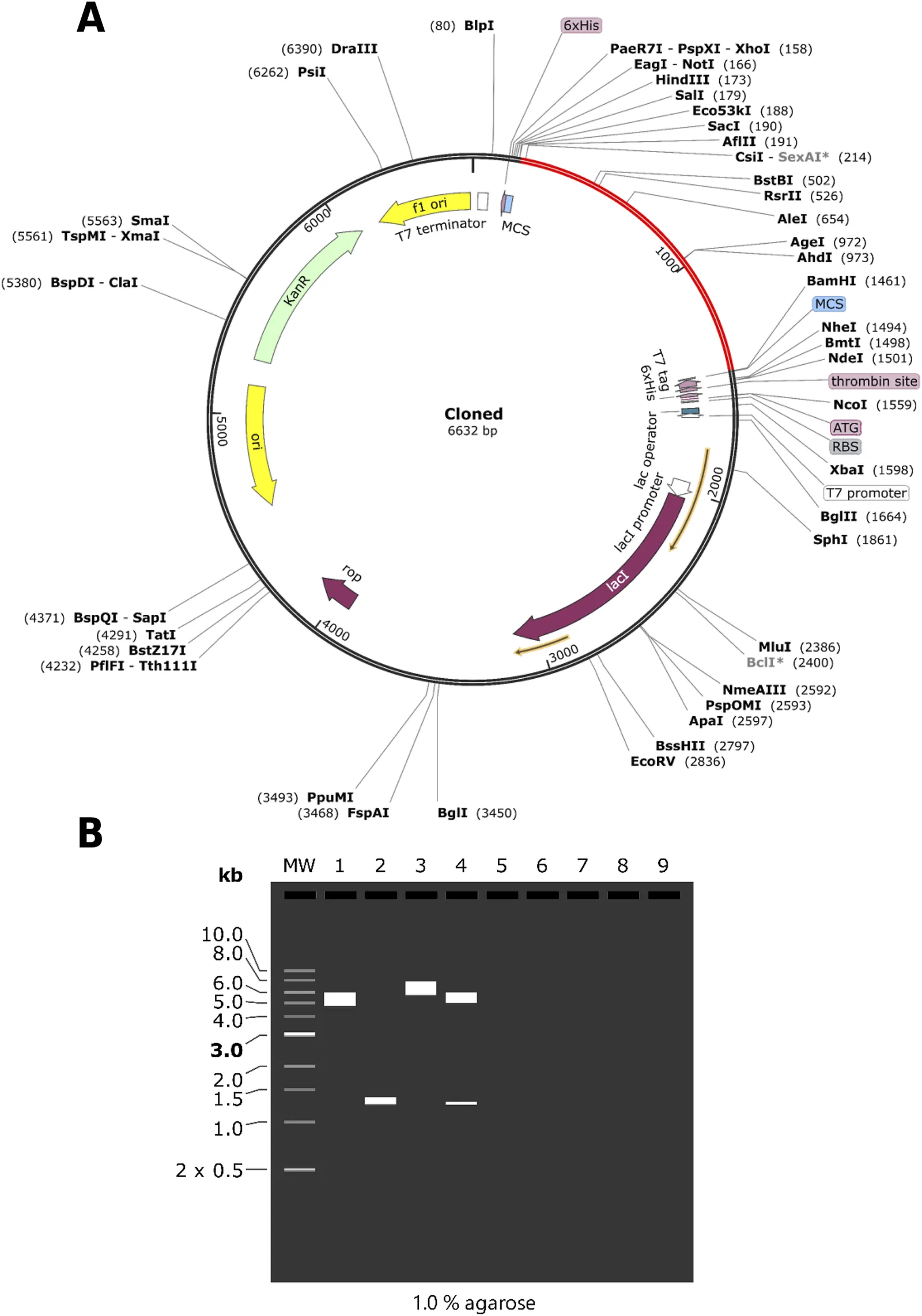
*In silico* cloning of the optimized vaccine construct into the pET-28a (+) expression vector. **(A)** The optimized vaccine sequence (red) was inserted into the multiple cloning site of the pET-28 (+) vector using the restriction enzymes SalI and BamHI. **(B)** *In silico* agarose gel electrophoresis (1.0%). Lane MW, molecular weight marker (MW); Lanes 1 pET-28a (+) vector (5,369 bp); Lane 2, optimized vaccine construct (1,271 bp); Lane 3 recombinant plasmid (6,632 bp); Lane 4, recombinant plasmid digested with SalI and BamHI, yielding fragments of 5,361 bp and 1,271 bp.

## 4 Discussion

Bacterial Kidney Disease (BKD) remains one of the most important infectious diseases affecting salmonid aquaculture worldwide and continues to represent a major sanitary and economic challenge, particularly in Chile, where its high prevalence has been associated with sustained and often ineffective antimicrobial use (Bayliss et al., 2018; Vargas-Chacoff et al., 2025). The development of effective vaccines against *R. salmoninarum* has historically been hindered by the pathogen’s ability to survive and replicate within host macrophages while actively modulating immune responses to evade bacterial clearance (Alcorn et al., 2005; Elliott et al., 2014). In this context, reverse vaccinology provides a rational framework for the identification of protective antigens and the design of targeted vaccine formulations. Accordingly, the present study describes the design and computational evaluation of RS-MEVC against *R. salmoninarum*.

A key feature of the proposed strategy was the deliberate exclusion of the major soluble antigen p57 (Msa). Although p57 is one of the most abundant and extensively studied proteins of *R. salmoninarum*, previous studies have demonstrated its immunomodulatory properties, including suppression of antibody production and inhibition of macrophage respiratory burst activity in salmonids (Brown et al., 1996; Fredriksen et al., 1997; Wiens et al., 1999; Aguilar et al., 2023). Therefore, the use of alternative virulence-associated proteins may reduce the risk of incorporating antigens that contribute to pathogen-mediated immune evasion while directing the immune response toward potentially protective determinants. Furthermore, the inclusion of proteomes derived from Chilean field strains increases the relevance of the selected antigens under local aquaculture conditions and may improve the applicability of the construct to regional production systems.

Because *R. salmoninarum* is a facultative intracellular pathogen, the induction of cellular immune responses is considered essential for effective protection (Zapata et al., 2006; Muñoz-Atienza et al., 2021). In teleost fish, resistance against intracellular bacteria relies on the coordinated activation of CD4^+^ and CD8^+^ T lymphocytes, generally associated with Th1-like immune profiles characterized by enhanced macrophage activation and cellular effector functions (Zou et al., 2005; Mokhtar et al., 2023). For this reason, epitope selection prioritized peptides predicted to bind multiple alleles of both MHC-I (UBA) and MHC-II (DAA/DAB) molecules (Zhao et al., 2008). This approach addresses the extensive polymorphism described for salmonid MHC loci and seeks to maximize population coverage within genetically diverse aquaculture stocks (Grimholt et al., 2003). However, an important limitation is that the MHC-I and MHC-II binding predictions were generated using NetMHCpan-4.1 and NetMHCIIpan-4.0, whose pan-specific models incorporate MHC sequence information but were developed predominantly from mammalian MHC–peptide binding and eluted-ligand datasets (Jurtz et al., 2017; Reynisson et al., 2020). Although this architecture allows predictions to be extended to MHC molecules for which limited allele-specific experimental data are available, the structural and functional characteristics of salmonid MHC molecules warrant caution in interpreting these predictions. Indeed, experimental studies have demonstrated distinct peptide-binding characteristics in *Atlantic salmon* MHC molecules, including the UBA locus (Zhao et al., 2008; Lukacs et al., 2010). In addition, the Score_EL ≥ 0.6 threshold used in this study should not be considered a salmonid-specific experimentally validated cutoff, and the original analysis did not include a systematic sensitivity analysis using alternative thresholds. Accordingly, the predicted binding of RS-MEVC epitopes should be interpreted as an *in silico* prioritization strategy rather than as experimental evidence of peptide–MHC binding. Experimental peptide–MHC binding assays and immunological studies in *A. salmon* will ultimately be required to confirm the specificity and biological relevance of the selected epitopes. Importantly, the predicted allelic breadth should not be interpreted as formal population coverage, as allele-frequency data for the specific *S. salar* MHC variants analyzed were not incorporated. In the absence of such data, MHC-I epitopes were prioritized primarily according to predicted allelic promiscuity, followed by antigenicity, whereas MHC-II candidates were prioritized according to antigenicity because all evaluated candidates exceeded the predefined binding threshold. Because high MHC-I promiscuity was concentrated in a limited number of candidates, increasing the number of epitopes may not substantially improve theoretical coverage without knowledge of population allele frequencies. The identification of linear B-cell epitopes was based on BepiPred-3.0 predictions followed by additional sequence and structural filtering. Nevertheless, computationally predicted B-cell epitopes should be regarded as candidate antigenic regions rather than experimentally validated immunogenic epitopes, as current computational approaches have limitations in accurately identifying antibody-recognized epitopes (Galanis et al., 2021; Clifford et al., 2022). Some predicted epitopes may not elicit antibody responses *in vivo* or may contribute differently to the overall immunogenicity of the multiepitope construct. Therefore, experimental validation of the selected B-cell epitopes will be necessary to determine their individual immunogenicity and contribution to vaccine-induced protection (Zheng et al., 2023). Consequently, the final construct was designed to promote both cellular and humoral immune responses while maintaining broad theoretical applicability across *A. salmon* populations. The selection of five epitopes per category was therefore intended to balance predicted immunological breadth with construct length and experimental tractability. Although increasing the number of epitopes could theoretically broaden allelic representation, the limited promiscuity observed among MHC-I candidates and the absence of representative allele-frequency data prevent predicting whether a larger epitope repertoire would meaningfully improve population coverage. A formal population coverage analysis and systematic comparison of alternative epitope sets will be required once representative salmonid allele-frequency data become available.

To further enhance immunogenicity, conserved flagellin-derived D0/D1 domains were incorporated as a molecular adjuvant. Flagellin is a well-characterized agonist of Toll-like receptor 5 (TLR5), whose activation stimulates innate immune responses and promotes the production of pro-inflammatory cytokines that contribute to the development of adaptive immunity (Tsujita et al., 2004; Qin et al., 2018; Wangkahart et al., 2019; Luo et al., 2019; Afzal et al., 2025). Importantly, TLR5 is structurally and functionally conserved in salmonids, supporting the biological rationale for its inclusion in the vaccine design (Ghani et al., 2024). In addition, the combination of rigid (EAAAK) and flexible (AAY, KK, and GPGPG) linkers was intended to facilitate appropriate epitope processing and presentation while preserving the structural integrity of the construct (Shamriz et al., 2016; Liu et al., 2024; Ma et al., 2024).

The structural and physicochemical analyses performed in this study further support the feasibility of the proposed vaccine design. The RS-MEVC construct exhibited favorable antigenicity, stability, and non-allergenicity profiles, which are desirable characteristics for recombinant vaccine development. Likewise, tertiary structure validation demonstrated excellent stereochemical quality, as evidenced by the Ramachandran plot analysis, which placed nearly all residues within favored regions, and by the ProSA Z-score, which was consistent with values reported for experimentally resolved proteins of comparable size (Shahbazi et al., 2025; Sharma et al., 2025). Together, these results support the predicted structural reliability of the construct and provide a basis for subsequent experimental characterization and recombinant expression. The optimized sequence showed a CAI of 0.89, indicating a high degree of adaptation to the codon usage of the target host (Sharp and Li, 1987). Nevertheless, CAI represents only one aspect of heterologous gene expression, and additional sequence features can influence translation efficiency. In particular, mRNA secondary structure around the translation initiation region can substantially affect ribosome accessibility and protein production in *E. coli* (Kudla et al., 2009). Thus, although the overall codon optimization was considered suitable for the computational design of RS-MEVC, further optimization of local sequence features could be explored if experimental expression proves suboptimal. The GC content of 59% should likewise be considered in this context, although global GC content alone is not sufficient to predict expression efficiency.

The predicted interaction between RS-MEVC and Atlantic salmon TLR5 provides additional support for the structural rationale underlying the construct. Molecular docking analyses identified an extensive interaction interface involving hydrogen bonds, salt bridges, and numerous non-bonded contacts, similar to interaction features reported for other *in silico* vaccine constructs (Masum et al., 2024; Sabzevari et al., 2025). While native flagellin–TLR5 interactions form higher-order assemblies, including the 2:2 arrangement observed in 3V47 and the multimeric assembly in 1U0A, initial receptor recognition involves a fundamental 1:1 interaction interface. To enable a direct comparison, we therefore isolated the primary 1:1 binding interface from the larger crystallographic assemblies. The resulting interaction networks showed comparable features, with the primary 1:1 interface in 3V47 and 1U0A exhibiting 14 and 18 hydrogen bonds, 2 and 1 salt bridges, and 181 and 182 non-bonded contacts, respectively. The corresponding RS-MEVC–TLR5 complex exhibited 14 hydrogens bonds, 5 salt bridges, and 206 non-bonded contacts. The similarity in these interaction features supports the structural plausibility of the predicted RS-MEVC–TLR5 interface, although it does not constitute experimental evidence of receptor engagement.

Furthermore, molecular dynamics simulations indicated that TLR5 association was accompanied by reduced backbone deviation and lower per-residue fluctuations of the RS-MEVC construct over the simulated timescale. The lower RMSD and RMSF values in the complex, together with the absence of a detectable difference in radius of gyration, suggest that receptor association reduces local and global conformational fluctuations without substantially altering the overall compactness of the construct. Consistent with these structural observations, MM-PBSA calculations yielded a negative predicted binding free energy, primarily driven by favorable electrostatic and van der Waals contributions. Although these computational predictions cannot replace experimental validation, they support the structural plausibility of the proposed TLR5–RS-MEVC interaction and provide a basis for further experimental investigation (Khan et al., 2021; Amaya-Ramirez et al., 2023; Guo et al., 2025).

Although the incorporation of the conserved D0/D1 domains of flagellin provides a rational strategy for TLR5-mediated innate immune stimulation (Yoon et al., 2012; Biedma et al., 2019), the safety and specificity of repeated exposure to a flagellin-based adjuvant warrant consideration. TLR5 recognizes conserved structural features of bacterial flagellin, and therefore potential interactions with flagellated members of the fish microbiota cannot be completely excluded. At the same time, the present construct contains only the conserved D0/D1 regions and lacks the hypervariable D2/D3 domains, which are major targets of the adaptive antibody response to flagellin. Their exclusion is therefore expected to reduce the intrinsic antigenicity of the flagellin-derived component while retaining the conserved regions required for TLR5 recognition (Yoon et al., 2012; Biedma et al., 2019). However, this design does not eliminate the possibility of functional TLR5 engagement by endogenous bacterial flagellins, and the consequences of repeated exposure to the recombinant D0/D1 construct on microbiota composition and mucosal homeostasis remain to be established.

Importantly, studies in salmonids provide a relevant precedent for the use of flagellin-derived molecules as immunostimulatory components. Flagellin stimulation in rainbow trout has been associated with rapid induction of pro-inflammatory mediators followed by attenuation of several responses within approximately 24 h, together with increased expression of soluble TLR5, suggesting the presence of regulatory mechanisms that may limit prolonged TLR5 signaling (Wangkahart et al., 2019). In Atlantic salmon, recombinant flagellin and its D1 domain have also been shown to induce immunostimulatory and pro-inflammatory responses and to enhance vaccine responses against *Piscirickettsia salmonis* (González-Stegmaier et al., 2021). These findings support the biological activity of flagellin-derived molecules in salmonids; however, they should not be interpreted as establishing the safety of repeated administration of the specific RS-MEVC construct. Future studies should therefore evaluate dose-dependent responses, repeated-dose effects, intestinal microbiota composition, mucosal homeostasis, and tissue integrity to establish the safety profile of RS-MEVC.

The bidirectional arrangement of the conserved flagellin domains, with D0/D1 at the N-terminus and D1/D0 at the C-terminus of RS-MEVC, was selected based on the structural organization of native flagellin and the established contribution of the conserved D0/D1 regions to TLR5 recognition and signaling (Yonekura et al., 2003; Song et al., 2017) This configuration was intended to retain the conserved terminal regions associated with TLR5 engagement while flanking the antigenic region. However, although this architecture provides a rational structural basis for the construct design, our computational analyses do not demonstrate that duplication of the D0/D1 module results in greater TLR5 engagement than a construct containing a single D0/D1 module. Therefore, the bidirectional configuration should be considered a rational design strategy rather than a demonstrated optimization of TLR5 agonism, and any potential functional advantage should be experimentally validated.

Beyond the predicted molecular interaction with TLR5, an important translational consideration for the further development of RS-MEVC is its route of administration. Given the chronic intracellular lifestyle of *R. salmoninarum* and its involvement of internal organs, including the kidney and spleen, the induction of effective systemic cellular immunity is an important consideration for vaccine development (Delghandi et al., 2020). Accordingly, intraperitoneal (IP) injection is proposed as the primary route for the initial experimental evaluation of RS-MEVC, as it provides controlled systemic delivery of the recombinant construct and is widely used in experimental vaccination studies in salmonids. This route is also commonly used for experimental vaccination studies in salmonids (Bøgwald and Dalmo, 2021). The inclusion of the conserved D0/D1 domains of flagellin as a TLR5-targeting molecular adjuvant provides an additional rationale for evaluating the capacity of the construct to induce systemic innate and adaptive immune responses (Biedma et al., 2019).

Nevertheless, oral and immersion vaccination remain attractive alternatives for aquaculture because of their potential for mass administration and reduced handling of fish. These mucosal delivery routes present additional challenges related to antigen stability, uptake, and the induction of effective mucosal and systemic immunity (Somamoto and Nakanishi, 2020). Therefore, if these approaches are pursued for RS-MEVC, future studies should evaluate appropriate formulation and delivery strategies, including encapsulation or particulate systems, to protect the recombinant antigen and enhance its uptake (Bøgwald and Dalmo, 2021). The optimal administration route, formulation, dose, and resulting immune profile will ultimately require experimental validation.

Regarding the molecular dynamics analyses, the 100 ns trajectories provide information on the short-term structural behavior of the predicted complexes but are insufficient to characterize slow binding/unbinding events, large-scale conformational rearrangements, or long-term complex stability. Although the favorable predicted MM-PBSA binding energy and reduced structural fluctuations support the short-term structural stability of the RS-MEVC–TLR5 complex, they provide only a limited view of its longer-timescale conformational landscape. Extended simulations at the microsecond scale, together with independent replicate trajectories, would therefore strengthen assessment of the reproducibility and long-timescale dynamics of the predicted interaction.

In addition, computational models cannot fully account for physiological and environmental variables that influence vaccine performance in aquaculture settings, including water temperature, nutritional status, stress, microbiota composition, and host genetic variability (Silva-Arrieta et al., 2020; Cernuto et al., 2025). Consequently, the immunogenicity and protective efficacy predicted here should be interpreted as preliminary and require experimental confirmation.

Collectively, these results support the potential of RS-MEVC as a promising vaccine candidate against BKD. The integration of conserved antigenic regions predicted B- and T-cell epitopes, a TLR5-targeting adjuvant strategy, favorable physicochemical and structural characteristics, and a computationally supported receptor interactions provides a solid foundation for its further development. Notably, the construct was designed to promote both humoral and cellular immune responses, the latter being particularly important for protection against intracellular pathogens such as *R. salmoninarum*.

The next logical step in the development of this strategy is experimental validation. Recombinant expression and purification of the construct should first be optimized, followed by targeted *in vitro* assays to test key prediction of the computational design. A TLR5 reporter assay would provide a direct first step to determine whether the flagellin D0/D1 domains incorporated into RS-MEVC activate the TLR5 pathway. This could be complemented by macrophage activation assays using salmonid macrophage-like cell lines such as SHK-1 or primary head kidney leukocytes (Stenberg et al., 2019; Muñoz et al., 2021). These studies could also evaluate immune recognition of the predicted MHC-I and MHC-II epitopes. Subsequently, *in vivo* vaccination trials will be necessary to characterize both humoral and cellular immune responses and to determine protective efficacy following challenge with virulent *R. salmoninarum* strains. Such studies should include evaluation of relative percent survival (RPS), bacterial burden in target organs, and immunological correlates of protection. These experiments will ultimately determine the practical value of RS-MEVC as a vaccine candidate for the control of BKD in aquaculture systems.

## 5 Conclusion

In this study, an RS-MEVC vaccine candidate against *R*. *salmoninarum* was designed using reverse vaccinology and immunoinformatic approaches. This strategy enabled the identification of conserved antigenic proteins shared among different bacterial strains and the prioritization of B-cell and T-cell epitopes with predicted immunological relevance for *S. salar*. The incorporation of flagellin-derived domains as a molecular adjuvant was designed to facilitate TLR5 mediated innate immune recognition and potentially enhance the immune responses to the vaccine construct.

The final construct, RS-MEVC, exhibited favorable physicochemical and structural characteristics, including antigenicity, stability, non-allergenicity, and acceptable stereochemical quality. Molecular docking and molecular dynamics simulations provided computational support for the structural plausibility and short-term stability of the predicted RS-MEVC-TLR5 interaction. However, these findings should not be interpreted as experimental evidence of receptor engagement or long-term complex stability. Similarly, the predicted MHC-I and MHC-II interactions represent an *in silico* prioritization strategy and do not establish experimentally validated peptide–MHC binding or population-level coverage in *A. salmon*. *In silico* codon optimization and cloning analyses further indicated the feasibility of recombinant expression in *E. col*i.

Overall, the present findings support RS-MEVC as a promising computationally designed vaccine candidate against bacterial kidney disease caused by *R. salmoninarum*. Experimental validation including recombinant expression, peptide–MHC binding and immunological assays, followed by vaccination and challenge studies in *A. salmon*, will be essential to determine its immunogenicity, safety, protective efficacy, and potential applicability under aquaculture conditions.

## Data Availability

The original contributions presented in the study are included in the article/Supplementary Material, further inquiries can be directed to the corresponding author.
