# Supplementary material for: Reverse vaccinology and immunoinformatics-driven design of a multiepitope vaccine candidate against Renibacterium salmoninarum
Source: Front Bioinform. 2026 Sep 25;6:1899416. doi: 10.3389/fbinf.2026.1899416 (PMC13650752; doi:10.3389/fbinf.2026.1899416)
Supplement: Supplementary file 1 [file Table1.docx]

**Table S1.** List of software tools and servers used in the study

| **Software/Server** | **Stage** | **Function** | **URL** |
| --- | --- | --- | --- |
| PSORTb | Protein selection | Subcellular localization prediction | <https://www.psort.org/psortb/index.html> |
| VFDB | Protein selection | Identification of virulence factors | <http://www.mgc.ac.cn/VFs/main.htm> |
| DEG | Protein selection | Identification of essential genes/proteins | <http://origin.tubic.org/deg/public/index.php> |
| BLASTp | Protein selection | Exclusion based on host homology | <https://blast.ncbi.nlm.nih.gov/Blast.cgi?PROGRAM=blastp> |
| TMHMM v2.0 | Protein selection | Transmembrane helix prediction | <https://services.healthtech.dtu.dk/services/TMHMM-2.0/> |
| VaxiJen v2.0 | Protein selection/ final evaluation | Antigenicity prediction | <https://www.ddg-pharmfac.net/vaxijen/VaxiJen/VaxiJen.html> |
| NetMHCpan-4.1 | Epitope prediction | MHC-I/CTL epitope prediction | <https://services.healthtech.dtu.dk/services/NetMHCpan-4.1/> |
| AllerTOP v2.0 | Safety assessment | Allergenicity prediction | <https://www.ddg-pharmfac.net/AllerTOP/> |
| ToxinPred2 | Safety assessment | Toxicity prediction | <https://webs.iiitd.edu.in/raghava/toxinpred2/> |
| BepiPred-2.0 | Epitope prediction | Linear B-cell epitope prediction | <https://services.healthtech.dtu.dk/services/BepiPred-2.0/> |
| NetMHCpan-4.0 | Epitope prediction | MHC-I epitope prediction | <https://services.healthtech.dtu.dk/services/NetMHCpan-4.0/> |
| ProtParam | Construct characterization | Calculation of physicochemical properties | <https://web.expasy.org/protparam/> |
| PDBsum | Structural analysis | Secondary structure and interaction analysis | <http://www.ebi.ac.uk/thornton-srv/databases/pdbsum/> |
| AlphaFold3 | Structural modeling | 3D structure prediction | <https://alphafoldserver.com> |
| PyMOL | Structural visualization | Visualization of 3D models | <https://pymol.org/> |
| GalaxyWEB | Structural modeling | Structural refinement | <https://galaxy.seoklab.org/> |
| MolProbity | Structural validation | Evaluation of geometry and atomic contacts | <http://molprobity.biochem.duke.edu/> |
| ProSA-web | Structural validation | Energetic validation of the model | <https://prosa.services.came.sbg.ac.at/prosa.php> |
| PROCHECK | Structural validation | Geometric validation of the model | <https://servicesn.mbi.ucla.edu/PROCHECK/> |
| ERRAT | Structural validation | Structural quality assessment | <https://servicesn.mbi.ucla.edu/ERRAT/> |
| ClusPro 2.0 | Molecular docking | Protein–protein docking | <https://cluspro.org/> |
| CHARMM-GUI | Molecular dynamics | System preparation for simulation | <https://www.charmm-gui.org/> |
| GROMACS v2025.1 | Molecular dynamics | Molecular dynamics simulation | <https://www.gromacs.org/> |
| gmxMMPBSA | Molecular dynamics | Binding free energy calculation | <https://valdes-tresanco-ms.github.io/gmx_MMPBSA/> |
| EMBOSS Backtranseq | Codon optimization | Reverse translation and initial optimization | <https://www.bioinformatics.nl/cgi-bin/emboss/backtranseq> |
| GenScript Rare Codon Analysis | Codon optimization | Evaluation of codon bias and expression | <https://www.genscript.com/tools/rare-codon-analysis> |
| SnapGene | *In silico* cloning | Construction and simulation of cloning | <https://www.snapgene.com/> |
| Addgene / pET-28a | *In silico* cloning | Expression vector used as backbone | <https://www.addgene.org/> |
